# Supplementary material for: A socio-ecological System Dynamics model of antimicrobial use and resistance
Source: PLoS One. 2026 Apr 20;21(4):e0347021. doi: 10.1371/journal.pone.0347021 (PMC13094956; doi:10.1371/journal.pone.0347021)
Supplement: S1 Table — (DOCX) [file pone.0347021.s001.docx]

# Supplementary table

Table S1. Settings used for sensitivity analysis

| **Parameter(s) assessed in sensitivity analysis** | **Parameter range explored** | **Values of other parameters** | **Sensitivity analysis settings** |
| --- | --- | --- | --- |
| a) timescale of microbial evolution and b) time horizon of clinician judgment (latter tested in anecdotal version only) | 0.1-26 weeks for timescale of microbial evolution  1-26 weeks for time horizon of clinician judgment (in anecdotal version only) | Initial susceptible fraction 1, relative fitness advantage in presence of antimicrobial 0.95, relative fitness cost in absence of antimicrobial 0.05, mean antimicrobial course length 2 weeks | Limited runs (Sobol sequencing); 50 runs; run duration 2080 weeks for anecdotal prescribing version; 520 weeks for surveillance variant. Additional 25 runs with timescale of microbial evolution at 0.1-2 weeks for surveillance. |
| Relative fitness a) advantage of resistance in presence of antimicrobial and b) cost of resistance in absence of antimicrobial | 1×10^-14^ to 1 for relative fitness under both conditions | Initial susceptible fraction 1, time horizon of clinician judgment 10 weeks (anecdotal prescribing variant only), timescale of microbial evolution 5 weeks, mean antimicrobial course length 2 weeks | Limited runs (Sobol sequencing); 50 runs; run duration 2080 weeks for anecdotal prescribing version; 520 weeks for surveillance. |
