# Supplementary figures and images for: A socio-ecological System Dynamics model of antimicrobial use and resistance
Source: PLoS One. 2026 Apr 20;21(4):e0347021. doi: 10.1371/journal.pone.0347021 (PMC13094956; doi:10.1371/journal.pone.0347021)

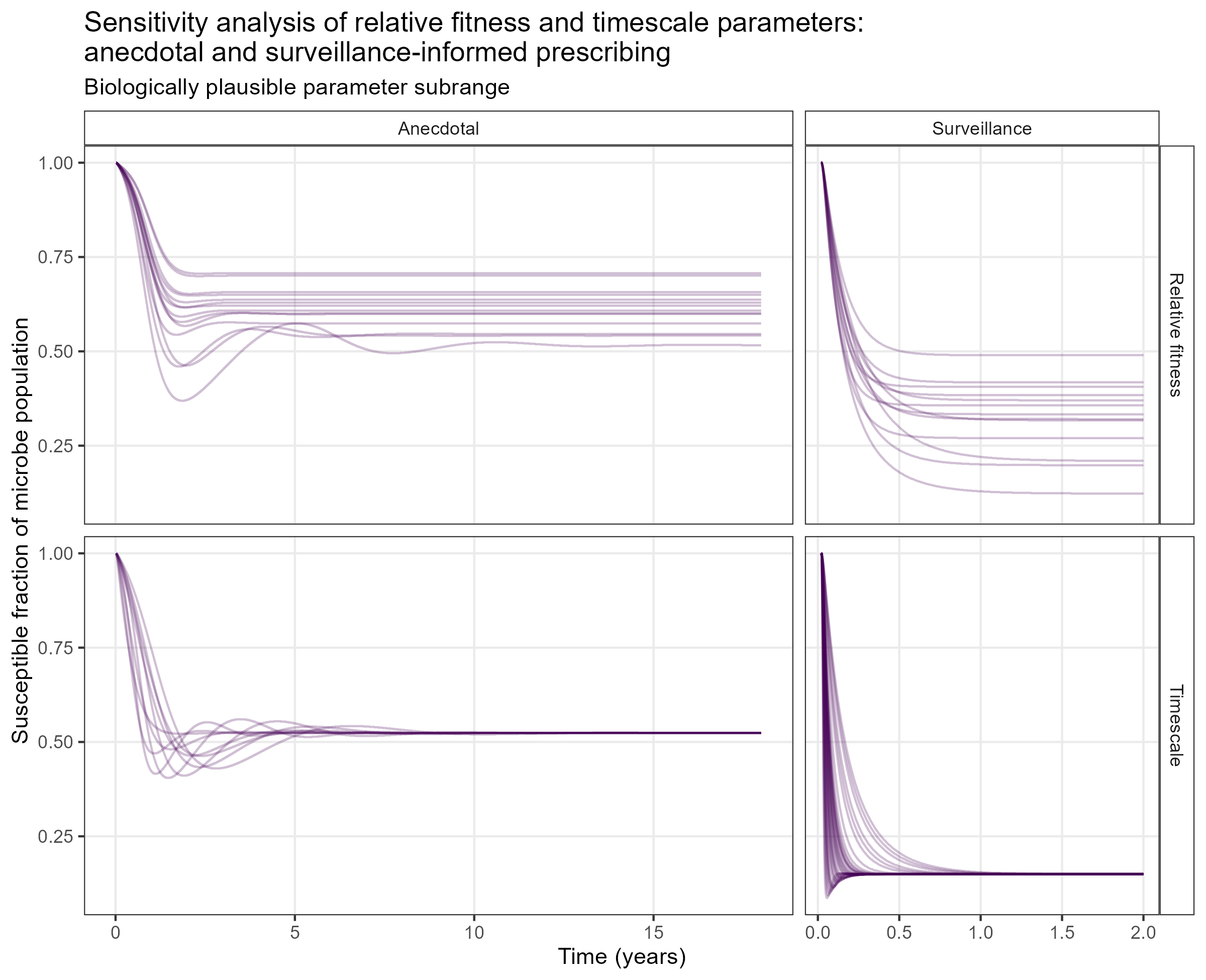

Supplement: S1 Fig — (TIF) [file pone.0347021.s002.tif]

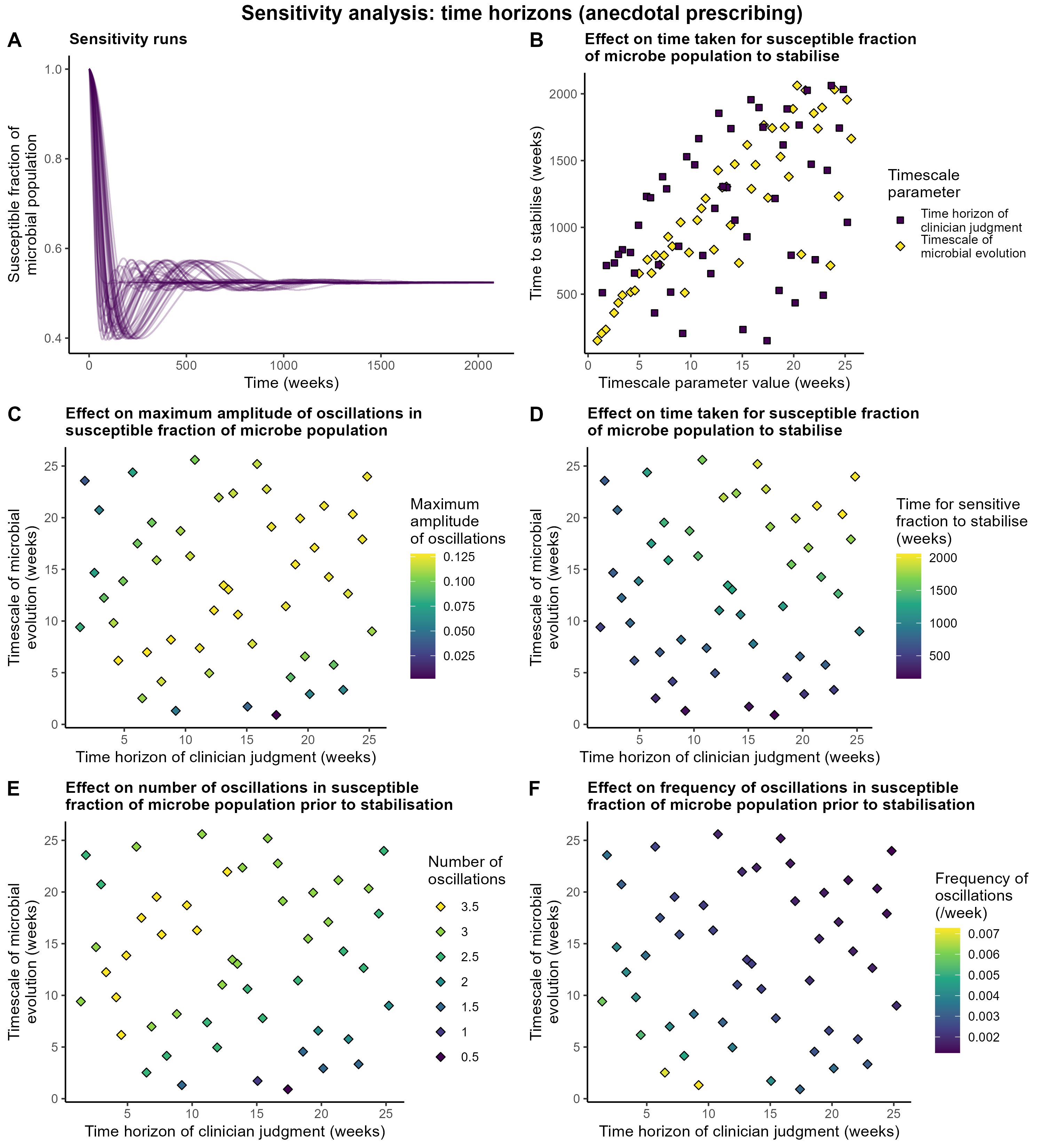

Supplement: S2 Fig — (TIF) [file pone.0347021.s003.tif]

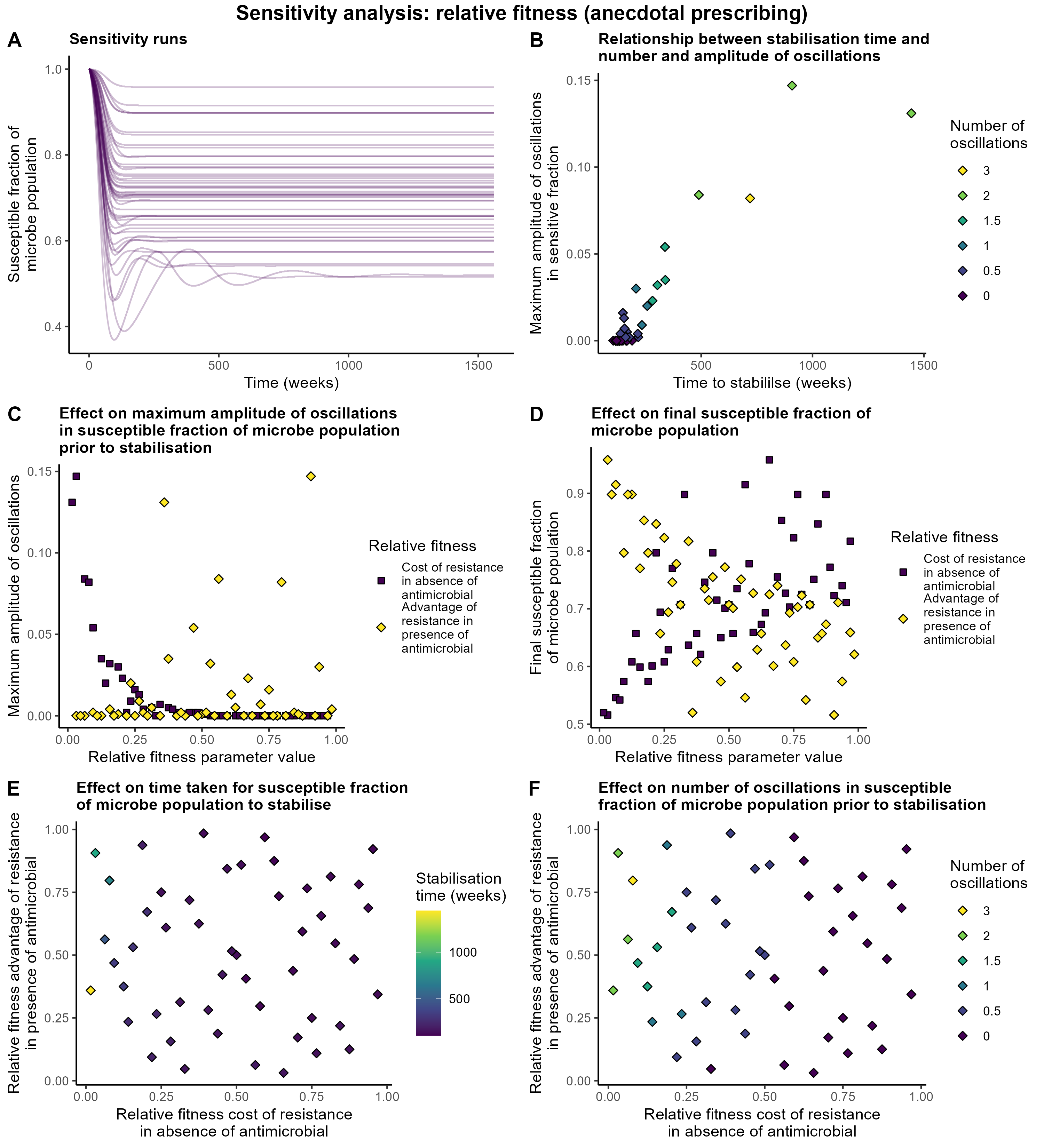

Supplement: S3 Fig — (TIF) [file pone.0347021.s004.tif]

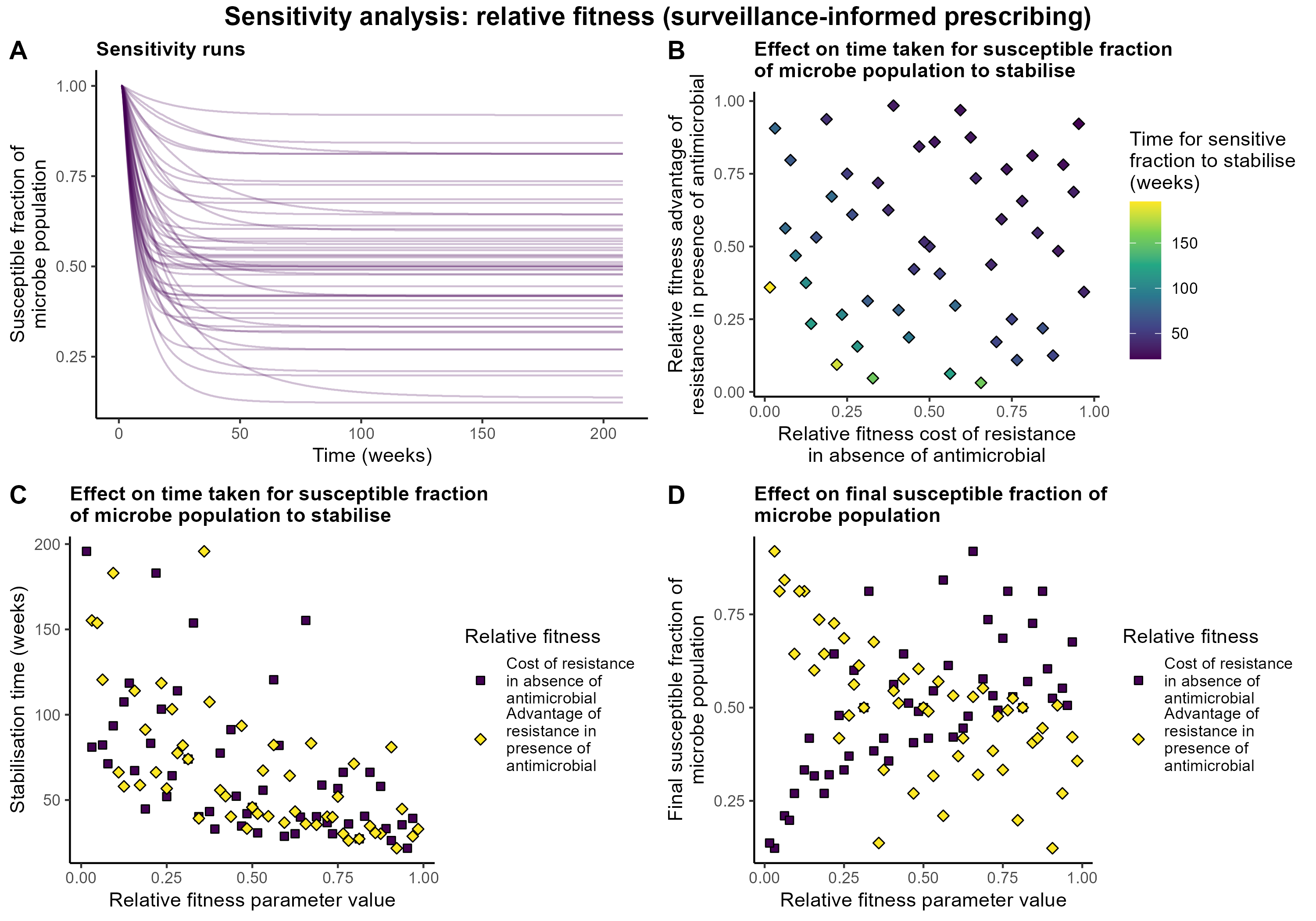

Supplement: S4 Fig — (TIF) [file pone.0347021.s005.tif]
