# Supplementary material for: A socio-ecological System Dynamics model of antimicrobial use and resistance
Source: PLoS One. 2026 Apr 20;21(4):e0347021. doi: 10.1371/journal.pone.0347021 (PMC13094956; doi:10.1371/journal.pone.0347021)
Supplement: S1 File — (DOCX) [file pone.0347021.s006.docx]

# Supplementary material

The supplementary material supplied for this paper are as follows:

- Model files with documentation (also available to download at <doi.org/10.6084/m9.figshare.31792000>)
  - AMR_LTG_anecdotal.stm (model from Fig. 3a)
  - AMR_LTG_surveillance.stm (model from Fig. 3b)
  - These Stella Architect files can be opened with isee Player (free software): <https://www.iseesystems.com/store/products/player.aspx>
- Sensitivity run outputs (.xls file)
- Settings used for sensitivity analysis (Table S1)
- Additional sensitivity run figures (Figures S1-S4)
