# Supplementary material for: A socio-ecological System Dynamics model of antimicrobial use and resistance
Source: PLoS One. 2026 Apr 20;21(4):e0347021. doi: 10.1371/journal.pone.0347021 (PMC13094956; doi:10.1371/journal.pone.0347021)
Supplement: Attachment — Submitted filename: Responses to reviewers.docx [file pone.0347021.s009.docx]

# Journal Requirements

When submitting your revision, we need you to address these additional requirements.

1.Please ensure that your manuscript meets PLOS ONE's style requirements, including those for file naming. The PLOS ONE style templates can be found at

<https://journals.plos.org/plosone/s/file?id=wjVg/PLOSOne_formatting_sample_main_body.pdf> and

<https://journals.plos.org/plosone/s/file?id=ba62/PLOSOne_formatting_sample_title_authors_affiliations.pdf>

*Response: Thank you for raising this; we have updated the formatting to match the journal requirements.*

2. We note that the grant information you provided in the ‘Funding Information’ and ‘Financial Disclosure’ sections do not match.

When you resubmit, please ensure that you provide the correct grant numbers for the awards you received for your study in the ‘Funding Information’ section.

*Response: We believe that these now match. The grants included in the financial disclosure are as follows: BBSRC grants* *BB/T00875X/1 (CRW), BB/Z515644/1 (DM), BB/T004436/1 (DM), BB/T004452/1 (DM) and Roslin Institute core funding (AM); University of Edinburgh Chancellor’s Fellowship (AM). All six of these grants (two of which do not have numbers) are listed in the funding sources list.*

3. Thank you for stating the following financial disclosure:

“CRW acknowledges support for a studentship part-funded by Zoetis and the UKRI Biotechnology and Biological Sciences Research Council (BBSRC) under grant number BB/T00875X/1. DM also acknowledges support under UKRI awards UKRI (BB/Z515644/1, BB/T004436/1 and BB/T004452/1, the latter of which is [co] funded by the UK Department of Health and Social Care as part of the Global AMR Innovation Fund (GAMRIF). This is a UK aid programme that supports early-stage innovative research in underfunded areas of antimicrobial resistance (AMR) research and development for the benefit of those in low- and middle-income countries (LMICs), who bear the greatest burden of AMR. The views expressed in this publication are those of the author(s) and not necessarily those of the UK Department of Health and Social Care. AM is funded by the University of Edinburgh Chancellor's Fellowship and BBSRC core funding for the Roslin Institute.”

Please state what role the funders took in the study.  If the funders had no role, please state: "The funders had no role in study design, data collection and analysis, decision to publish, or preparation of the manuscript."

If this statement is not correct you must amend it as needed.

Please include this amended Role of Funder statement in your cover letter; we will change the online submission form on your behalf.

*Response: Thank you – this has been included in the cover letter.*

4. Thank you for stating the following in the Acknowledgments Section of your manuscript:

“CRW acknowledges support for a studentship part-funded by Zoetis and the UKRI Biotechnology and Biological Sciences Research Council (BBSRC) under grant number BB/T00875X/1. DM also acknowledges support under UKRI awards UKRI (BB/Z515644/1, BB/T004436/1 and BB/T004452/1, the latter of which is [co] funded by the UK Department of Health and Social Care as part of the Global AMR Innovation Fund (GAMRIF). This is a UK aid programme that supports early-stage innovative research in underfunded areas of antimicrobial resistance (AMR) research and development for the benefit of those in low and middle-income countries (LMICs), who bear the greatest burden of AMR. The views expressed in this publication are those of the author(s) and not necessarily those of the UK Department of Health and Social Care. AM is funded by the University of Edinburgh Chancellor's Fellowship and BBSRC core funding for the Roslin Institute. For the purpose of open access, the authors have applied a Creative Commons Attribution (CC BY) licence to any Author Accepted Manuscript version arising from this submission.”

We note that you have provided funding information that is not currently declared in your Funding Statement. However, funding information should not appear in the Acknowledgments section or other areas of your manuscript. We will only publish funding information present in the Funding Statement section of the online submission form.

Please remove any funding-related text from the manuscript and let us know how you would like to update your Funding Statement. Currently, your Funding Statement reads as follows:

“CRW acknowledges support for a studentship part-funded by Zoetis and the UKRI Biotechnology and Biological Sciences Research Council (BBSRC) under grant number BB/T00875X/1. DM also acknowledges support under UKRI awards UKRI (BB/Z515644/1, BB/T004436/1 and BB/T004452/1, the latter of which is [co] funded by the UK Department of Health and Social Care as part of the Global AMR Innovation Fund (GAMRIF). This is a UK aid programme that supports early-stage innovative research in underfunded areas of antimicrobial resistance (AMR) research and development for the benefit of those in low- and middle-income countries (LMICs), who bear the greatest burden of AMR. The views expressed in this publication are those of the author(s) and not necessarily those of the UK Department of Health and Social Care. AM is funded by the University of Edinburgh Chancellor's Fellowship and BBSRC core funding for the Roslin Institute.”

Please include your amended statements within your cover letter; we will change the online submission form on your behalf.

*Response: Thank you, this has been removed from the manuscript and is included in the cover letter.*

5. We are unable to open your Supporting Information file “Model files.zip”. Please kindly revise as necessary and re-upload.

*Response: Thank you for identifying this. We have uploaded a new version of the zip file and uploaded the individual model files to FigShare at* [*doi.org/10.6084/m9.figshare.31792000*](https://uoe-my.sharepoint.com/personal/s2252236_ed_ac_uk/Documents/Documents/PhD/Publishing/Socio-ecological%20model/Model%20structure%20and%20sensi%20analysis%20paper/Revisions/doi.org/10.6084/m9.figshare.31792000) *. Please note these model files require the free isee Player software to open and run, available from* [*https://www.iseesystems.com/store/products/player.aspx*](https://www.iseesystems.com/store/products/player.aspx)*.*

6. If the reviewer comments include a recommendation to cite specific previously published works, please review and evaluate these publications to determine whether they are relevant and should be cited. There is no requirement to cite these works unless the editor has indicated otherwise. 

Additional Editor Comments:

Please address the comments.

*Response: We have addressed and responded to all reviewer comments, which we feel have substantially improved the paper. We hope that you will find the revised manuscript acceptable.*

# Reviewers’ Comments

## Reviewer #1

The manuscript frames AMR as a Limits-to-Growth system and develops two complementary causal-loop diagrams (CLDs) and quantitative system-dynamics (SD) models: an anecdotal prescribing variant in which antimicrobial use (AMU) is driven by recent anecdotal success, and a surveillance-informed variant in which measured susceptibility feeds back to prescribing. The authors explore model behaviour across wide parameter ranges (microbial evolution timescales, clinician judgment horizon, and resistant fitness) with Sobol-sequence sampling and long runs (up to 40 years) to compare equilibria, oscillations, and time-to-stabilization between structures.
1. Casting AMR in an LTG frame is conceptually apt: AMU “harvests” susceptibility as a limiting resource and feedbacks to prescribing behaviour can generate oscillations and long transients. This framing will resonate with policy audiences and systems-modelling readers.
2. The contrast between anecdotal prescribing and surveillance-informed prescribing captures an important policy question — whether information feedbacks shorten harmful oscillatory dynamics and accelerate system stabilization.
3. The authors explore a wide parameter space (timescales, fitness) and report on qualitative features (oscillations, stabilisation time, equilibrium susceptible fraction), which is appropriate for exploratory SD work.
4. The finding that surveillance can dramatically reduce time to stabilisation is potentially policy-relevant and motivates investment in surveillance infrastructure.

Comments
1. The model treats AMU (antimicrobial use/effort) as exogenous or driven only by recent anecdotal success or by surveillance. The manuscript acknowledges this limitation but does not adequately justify treating AMU as independent of socio-behavioural drivers (costs, access, incentives, patient demand, prescribing norms), which are central determinants of AMR trajectories in real settings. Either (a) explicitly justify why a simplified AMU driver is appropriate for the questions posed (i.e., focusing on feedback form rather than full behavioural realism), or (b) extend the CLD/SD structure to include a simple behavioural module (e.g., prescribing incentives, patient demand, drug availability) and test sensitivity. At minimum, discuss how exclusion of these factors affects interpretation and policy translation.

*Response: Thank you for raising this. We have added explicit justification for the simplification (line 154) and added a paragraph in the discussion section addressing the implications of this choice for interpretation (line 417).*

2. The SD models appear entirely theoretical — parameter ranges are wide and not calibrated to empirical settings. Without calibration, it is hard to assess whether the oscillatory regimes or stabilization times are realistic for real pathogens/contexts. Provide at least one calibration/validation example using empirical data (even qualitative) — e.g., match model behaviour to known historical AMR dynamics for a pathogen/antimicrobial pair, or show how parameter choices would map to a plausible pathogen (e.g., E. coli, S. aureus) with references for mutation rates and fitness costs. If empirical calibration is infeasible, clearly label the study as conceptual and include guidance on how to adapt parameters for applied scenarios.

*Response: The model has now been labelled as conceptual (lines 17, 270, 288, 465) as the mechanistic representation of microbial ecology and evolution in the model presented here is not directly comparable with the usual Malthusian fitness parameters measured empirically. This has been added to the Discussion section (line 377), and we have added a brief discussion of how the evolutionary portion of the model would need to be configured to allow for calibration with empirical data (line 383).*

3. Parameter ranges (e.g., relative fitness from 1×10-14 to 1, microbial evolution timescale 0.1–26 weeks) are very broad; however, biological plausibility for extremes is not discussed. Some extremes (1×10-14) are effectively zero and may cause numerical issues. Justify choice of ranges with citations or sensitivity rationale. Consider restricting ranges to biologically plausible bounds for typical bacterial pathogens, and include a rationale for exploring extreme values (e.g., to test numerical stability). Report sensitivity results both across the full exploratory range and within a biologically plausible subrange.

*Response: Justification for the extreme parameter ranges has been added (lines 192-206): they allow for both testing of numerical stability of the model and exploration of model behaviour across a wide parameter space when calibration to empirically reported data is not feasible. In the case of relative fitness, these parameters are represented as replacement rates so could plausibly be functionally zero. We have included an additional figure plotting sensitivity results over a restricted parameter range based on biological plausibility (Fig S1).*

5. The current deterministic SD approach may understate stochastic effects important when resistant alleles are at very low frequency, or when population sizes are small. The manuscript discusses low initial gene frequency but implements deterministic reversion processes. Discuss limitations of deterministic modelling in representing stochastic emergence/extinction. Consider supplementing with stochastic simulations (Gillespie or stochastic SD) for scenarios where resistant allele frequencies are rare. Alternatively, include heterogeneity in mutation rates/fitness across strains as an extended sensitivity analysis.
*Response: We recognise this and have added a discussion of the limitations and implications of deterministic modelling (line 442) and added detail to the exploration of future development possibilities the model (line 448).*

This manuscript offers a clear and compelling systems framing of AMR dynamics and provides useful insights into how information feedback (surveillance) can alter system behaviour relative to anecdotal prescribing. The modelling approach and sensitivity exploration are valuable. However, before publication in a high-impact journal, the manuscript needs stronger empirical grounding, clearer model specification, and explicit treatment of behavioural drivers and stochastic effects that shape real-world AMR dynamics. Addressing these points will substantially increase the rigor, credibility, and policy relevance of the work.

*Response: Thank you for your positive feedback and constructive recommendations, which we feel have much improved the manuscript. We have added discussion of the implications of the model’s elision of further behavioural drivers and stochastic effects. We have also addressed the empirical grounding, including multiple real-life examples, and have identified ways in which the model, which is conceptual in its current form, could be adapted for calibration against empirical data. We hope we have addressed all comments to your satisfaction.*

## Reviewer #2

This manuscript presents a conceptually interesting and potentially valuable application of System Dynamics (SD) to antimicrobial resistance (AMR), framing antimicrobial susceptibility as a partially renewable natural resource and embedding human prescribing behaviour within a Limits-to-Growth (LTG) archetype. The behavioural interpretation of AMR plateaux is original and thought-provoking. However, in its current form, the paper does not yet meet the standards for methodological clarity, validation, and interpretability required for publication, even in a methods-oriented journal such as PLOS ONE. Substantial revision is required. I ivite the authors to clarify the following concerns:

1. The framing of susceptibility as a renewable or partially renewable natural resource is repeatedly highlighted as a key contribution. However, the manuscript does not clearly specify under what biological conditions susceptibility is truly renewable, versus quasi-renewable or effectively non-renewable. Fitness costs are treated as abstract parameters, but no clear biological mapping is provided (e.g., plasmid-borne resistance, compensatory evolution, clonal replacement). I invite the authors to add a short conceptual subsection explicitly defining what qualifies susceptibility as renewable in this model, which real-world AMR scenarios this framing plausibly applies to and which important AMR scenarios it explicitly does not apply to. Likewise, please provide at least two concrete biological examples (e.g., fluoroquinolone resistance in E. coli vs. ESBL plasmids) to anchor the abstraction. Without this, the “resource extraction” framing risks appearing metaphorical rather than analytical.

*Response: Thank you for highlighting this. We have added explanation of parameters (line 267) and added a conceptual section in the Methods section discussing renewability with several concrete biological examples (line 162).*

2. The SD structure assumes a single bug–drug–mutation–context, homogeneous host population, no environmental or between-host reservoirs and AMU determined either by anecdotal success or population-level surveillance. While simplification is acceptable, the rationale for these specific simplifications is not adequately defended. The authors must explicitly justify why anecdotal confidence is modelled as a smooth stock variable rather than, for example: threshold-based switching, policy-driven constraints and diagnostic-driven decisions.
*Response: A new subsection identifying, discussing, and justifying the assumptions (including those specifically mentioned in points 2 and 3) has been added in the Results section (line 269). Consideration of alternative modelling choices has been included in the Discussion section (lines 406, 425, 448).*

3. The mathematical formulation lacks clarity and biological interpretability. The system of equations (Equations 1–7) is central, yet several issues remain: equation 1–2: The symmetry between S and R is mathematically neat but biologically strong; mutation, reversion, and replacement are conflated; equation 5a/5b: The prescribing function is not intuitive, and the role of HE as a “time unit of prescribing” is unclear; equation 6: “Cases successfully treated” are defined as C=E×SC = E \times SC=E×S, implicitly assuming: perfect treatment efficacy for susceptible infections and zero efficacy for resistant infections Please provide a table mapping each equation term to a biological or behavioural process, explicitly state all implicit assumptions (perfect efficacy, no partial resistance, no treatment failure unrelated to AMR), clarify why S is equated to treatment success probability, and discuss implications and consider moving full equations to Supplementary Information and simplifying the main text, but with improved explanation.
*Response: We agree; a table of variables and their explanations has been added (line 269) to allow for clearer explanation, as has a section explicitly stating all assumptions and their implications, which are further explored in the Discussion section (line 389). The names of the relative fitness parameters have been modified for clarity and accuracy (“relative fitness advantage of resistance in presence of antimicrobial” and “relative fitness cost of resistance in absence of antimicrobial”) and differences between these and Malthusian parameters are explored in the Discussion section (line 377).*

4. The manuscript argues that human behavioural feedback alone could explain observed AMR plateaux, a strong and interesting claim. However, the model is not calibrated to any empirical dataset, no comparison is made with known evolutionary explanations beyond citation and the conclusion risks being interpreted as causal rather than exploratory. I invite the authors to reframe conclusions to emphasise hypothesis generation, not explanation, explicitly state that behavioural mechanisms are one possible contributor among many and add a paragraph outlining what empirical tests would be required to distinguish behavioural from evolutionary plateau mechanisms. This reframing is essential to avoid overinterpretation.

*Response: Thank you for pointing this out; we have now edited to emphasise hypothesis generation (lines 31, 349 and 480) and to include a discussion of how behavioural and evolutionary mechanisms could be distinguished (line 353).*

*Thank you for your helpful and constructive comments, which we feel have substantially improved the quality of the manuscript. We hope we have addressed all comments to your satisfaction.*
